# Prevalence of color vision deficiency in Africa: Systematic review and meta-analysis

**DOI:** 10.1371/journal.pone.0313819

**Published:** 2024-12-04

**Authors:** Mikias Mered Tilahun, Faisel Dula Sema, Berihun Aging Mengistie, Nardos Hussen Abdulkadir, Abdisa Gemedi Jara

**Affiliations:** 1 Department of Optometry, School of Medicine, College of Medicine and Health Science, Comprehensive Specialized Hospital, University of Gondar, Gondar, Ethiopia; 2 Department of Clinical Pharmacy, School of Pharmacy, College of Medicine and Health Sciences, University of Gondar, Gondar, Ethiopia; 3 Department of General Midwifery, School of Midwifery, College of Medicine and Health Sciences, University of Gondar, Gondar, Ethiopia; 4 Department of Occupational Therapy, School of Medicine, College of Medicine and Health Science, Comprehensive Specialized Hospital, University of Gondar, Gondar, Ethiopia; Marshall B. Ketchum University, UNITED STATES OF AMERICA

## Abstract

**Background:**

Color vision deficiency (CVD) cause is the difficulty distinguishing colors, which can present vocational and avocational challenges. There is a lack of data on its overall prevalence of CVD. Therefore, this systematic review and meta-analysis aim to determine the prevalence of CVD in Africa.

**Methods:**

The protocol was registered with the Prospective Register of Systematic Reviews (PROSPERO) database (protocol registration number: CRD42024510403). A comprehensive systematic literature search was conducted via PubMed/MEDLINE/EMBASE, Google, and Google Scholar from February 2024 to May 28, 2024. The Johanna Bridges Institute quality appraisal tool was used to assess the quality of eligible articles. The pooled prevalence of CVD among Africans was estimated using a random effect model and expressed as prevalence and odds ratios with 95% confidence intervals using Der Simonian-Laird weight. The I^2^ statistic test was used to measure heterogeneity, and subgroup analysis was performed based on country, source of population, and gender.

**Result:**

A total of 502 initial studies were identified, and sixteen cross-sectional studies were included. The overall pooled prevalence of CVD in Africa was 2.71% (95% CI: 2.28,3.14, I^2^ = 72.6%, P<0.001). The prevalence among African males and females was 2.13% and 0.34%, respectively. The highest pooled prevalence was recorded in Ethiopia at 3.63% and the prevalence among primary and secondary school students was 2.96%. A funnel plot showed that all of the studies were symmetric, and the Egger test showed no publication bias.

**Conclusion:**

The pooled prevalence of color vision deficiency in Africa was found to be 2.71%. The highest prevalence was reported in studies conducted among school-age children in Ethiopia. Establishing effective screening programs and raising public awareness are recommended as future steps.

## Introduction

Color vision deficiency (CVD) is the inability or decreased ability to perceive color differences [1, 2]. The condition primarily affects males due to its X-linked inheritance pattern [3, 4], but it can also occur as a result of an ocular, neurologic, or systemic cause [5]. CVD is one of the most common eye disorders worldwide [3].

The prevalence and distribution of CVD vary significantly across the global population and geographic regions. Individual reports from North America have indicated a range of 1.4% [6] –29% [7] of CVD, while studies from Europe have reported CVD levels ranging from 0.05% [8]–7.33% [9]. Studies from Asia have indicated a range of 1.17% [10]- 6.8% [11]. However, data on CVD in Africa are limited and fragmented, with reported prevalence levels ranging from 1.2% [4] to 4.84% [12].

Individuals affected by CVD face restrictions in performing color-guided tasks, leading to difficulty in daily activities [13] and challenges in professions requiring precise color discrimination, such as driving, military service, piloting, air traffic control, and healthcare roles [14–16].

The socioeconomic impact of CVD in Africa is exacerbated by lack of awareness among the African population about CVD [17, 18], inadequate screening programs for early detection of the condition [19], limited coping options for those affected [17], and a lack of comprehensive data on its prevalence and distribution in Africa. Therefore, conducting a systematic review and meta-analysis of the pooled prevalence of CVD in Africa is important to understand its prevalence, identify populations at higher risk, guide the development of public health strategies, contribute to the global knowledge about CVD, support advocacy, and increase awareness about CVD.

## Methods

### Reporting

This systematic review and meta-analysis were conducted to compile evidence published on CVD in Africa, and the protocol was registered in the Prospective Register of Systematic Reviews (PROSPERO) database (protocol registration number: CRD42024510403). The review followed the Preferred Reporting Items for Systematic Review and Meta-Analysis (PRISMA) guidelines [20] (S1 Checklist).

### Study selection and search strategy

This review included all studies that reported on the prevalence of CVD in African countries. Participants in the studies included individuals of any race, gender, or age residing in Africa. A comprehensive systematic literature search was conducted in PubMed/MEDLINE/Embase, Google, and Google Scholar, regardless of publication timelines, from February 2024 to May 28, 2024, as no systematic review and meta-analysis on CVD in Africa had been conducted.

The key search terms included “magnitude, prevalence, level, incidence, color/color vision deficiency, color impairment, color blindness, color perception defect, and color/colour vision defect” combined with the Boolean operators "AND" and "OR". For the PubMed/MEDLINE advanced search strategy, the advanced search strategy used was (((((((magnitude[Title/Abstract]) OR (Prevalence[Title/Abstract])) OR (Level[Title/Abstract])) OR (Incidence[Title/Abstract]))) AND (((("color vision deficiency"[Title/Abstract]) OR ("color impairment"[Title/Abstract])) OR ("color blindness"[Title/Abstract])) OR ("color defect"[Title/Abstract])) OR ("color perception defect"[Title/Abstract]))) OR ("colour vision deficiency"[Title/Abstract])) OR ("colour vision defects"[Title/Abstract]). In addition to the electronic database search, literature published in PubMed unindexed journals and gray literature search was conducted using direct Google Search and Google Scholar.

### Inclusion criteria

This systematic review and meta-analysis included articles that followed the Coco Pop mnemonic (Condition, Context, and Population) approach [21] and included studies of distinct levels of CVD published in English until May 28, 2024, in Africa.

### Exclusion criteria

Studies that did not meet the minimum quality assessment, lacked full access, or were focused on unrelated topics were excluded from this systematic review and meta-analysis.

### Study selection

Three review authors (MMT, AGJ, and NHA) independently screened articles based on their titles and abstracts. The identified articles were then combined, exported, and managed using Endnote X9.2 (Thomson Reuters, Philadelphia, PA, USA) software [22]. After duplicate studies were excluded, full-text appraisal was done by review authors (AGJ, BAM, and FDS), and the disagreement between authors during abstract and full-text selection were solved based evidence-based discussion and the involvement of the remaining review authors (MMT).

#### Outcome measurement

The primary outcome was the prevalence of CVD in Africa, which shows the number of people who had CVD using different screening methods or tools (Ishihara, Color Vision Testing Made Easy (CVTME), and Richmond-HRR (Hardy-Rand-Rittler)).

#### Data extraction

Three review authors (MMT, AGJ, and FDS) extracted the data independently using a Microsoft Excel spreadsheet. The differences among the three review authors were resolved through discussion and agreement. Any discrepancies were resolved after the other authors’ review (NHA and BAM). Disagreements were resolved based on a Kappa statistic threshold of 0.8, which was used to ensure substantial agreement among reviewers. The first author’s name, the year of publication, the year of study, the study country, the study design, the sample size, the source population, the age difference among participants, the technique used to assess CVD, the type of CVD assessed, the prevalence of CVD, the prevalence CVD among gender, and the factors associated with CVD were extracted from each study. We derived estimates from each study, and when needed, variables were not directly reported. The full data extraction sheet in detail is available in S1 Table.

#### Quality assessment

The Johanna Bridges Institute (JBI) quality appraisal tool for cross-sectional studies, a methodological quality assessment tool with nine questions, was used to evaluate the quality of included articles and the risk of bias in each study [23]. Two authors (MMT and AGJ) independently evaluated the quality of the included articles. The assessment tool contains nine criteria: It was assessed using the JBI critical appraisal checklist options of "yes," "no," "unclear," and "not applicable." The weights of yes, no, and unclear were 1, 0, and 0, respectively. Bias risks were classified as low (5 to 9) or high (0 to 4). The study received a 50% or higher rating on all quality-assessed items, which were deemed low-risk and included in this review. Disagreements that occurred during the full-text quality assessment were resolved through evidence-based discussion with the other review authors (FDS, NHA, and BAM) The result of quality assessment/risk of bias are presented in Table 1 and S2 Table.

**Table 1:** JBI methodological guidance for systematic reviews of observational epidemiological studies included in this systematic review and meta-analysis,2024.

Authors	Q1	Q2	Q3	Q4	Q5	Q6	Q7	Q8	Q9	Risk of bias
Gudeta and Asrat et al., [12]	Yes	No	Yes	Yes	Yes	Yes	Yes	No	Yes	Low
Darge et al., [24]	U/c	Yes	Yes	Yes	U/c	U/c	U/c	Yes	Yes	Low
Mashige et al., [25]	Yes	Yes	Yes	Yes	Yes	Yes	Yes	Yes	Yes	Low
Dohvoma et al., [26]	No	No	Yes	Yes	Yes	Yes	Yes	Yes	Yes	Low
Wale et al., [27]	Yes	Yes	Yes	Yes	Yes	U/c	Yes	No	Yes	Low
Mitiku et al, [28]	Yes	U/c	U/c	Yes	Yes	Yes	U/c	Yes	Yes	Low
Ugalahi et al., [29]	Yes	Yes	Yes	Yes	Yes	Yes	U/c	No	Yes	Low
Oduntan et al.,[17]	Yes	Yes	Yes	Yes	Yes	Yes	Yes	Yes	Yes	Low
Tabansi et al., [30]	Yes	No	Yes	Yes	Yes	Yes	Yes	No	Yes	Low
Woldeamanuel et al., [18]	Yes	Yes	Yes	Yes	Yes	Yes	Yes	No	Yes	Low
Mulusew et al., [31]	Yes	No	Yes	Yes	Yes	Yes	U/c	Yes	Yes	Low
Eze et al., [4]	Yes	Yes	U/c	Yes	U/c	Yes	Yes	Yes	U/c	Low
Mengesha et al., [32]	No	Yes	Yes	Yes	Yes	Yes	U/c	Yes	Yes	Low
Fakorede et al, [33]	Yes	U/c	Yes	Yes	Yes	U/c	Yes	U/c	Yes	Low
Ativie et al., [34]	Yes	Yes	Yes	Yes	Yes	Yes	U/c	No	Yes	Low
Nwobodo et al., [35]	Yes	Yes	No	Yes	Yes	Yes	U/c	No	Yes	Low

Q1; was the sample frame appropriate to address the target population? Q2 = were study participants sampled in an appropriate way? Q3; was the sample size adequate? Q4; were the study subjects and the setting described in detail? Q5; was the data analysis conducted with sufficient coverage of the identified sample? Q6; were valid methods used for the identification of the condition? Q7; was the condition measured in a standard, reliable way for all participants? Q8; was there appropriate statistical analysis? Q9; was the response rate adequate, and if not, was the low response rate managed appropriately? U/c; unclear

## Data analysis

The data was extracted using a Microsoft Excel spreadsheet and then exported to STATA version 11 for further analysis. Since the heterogenicity of this systematic review and meta-analysis is significant (I^2^ = 72.6%, P<0.001), the overall pooled prevalence of CVD among Africans was estimated using a random effect model and measured as prevalence and odds ratios with 95% confidence intervals using Der Simonian-Laird weight [36, 37]. The result was presented using tables and figures. Furthermore, the I^2^ statistic test was used to determine heterogeneity among the included studies, which describes the percentage of total variation caused by heterogeneity rather than chance. The forest plots were used to display the estimates and 95% confidence intervals from each individual study. In the meta-analysis, we used the inverse variance method to assign weights to each study. Sub-group analysis was performed based on the country in which the studies were conducted, gender, and the source of the population. We employed a funnel plot to detect publication bias, which allows us to assess whether smaller studies are more inclined to report extreme results. In addition, we conducted Egger’s test to statistically evaluate the symmetry observed in the funnel plot. To determine the impact of individual studies on the pooled estimate, a sensitivity analysis was performed. Missing data was handled by excluding the studies that missed the pertinent data and available case analysis was used. In addition, when needed, variables not reported directly, we derived estimates from each study.

## Result

### Study selection

**Fig 1:**
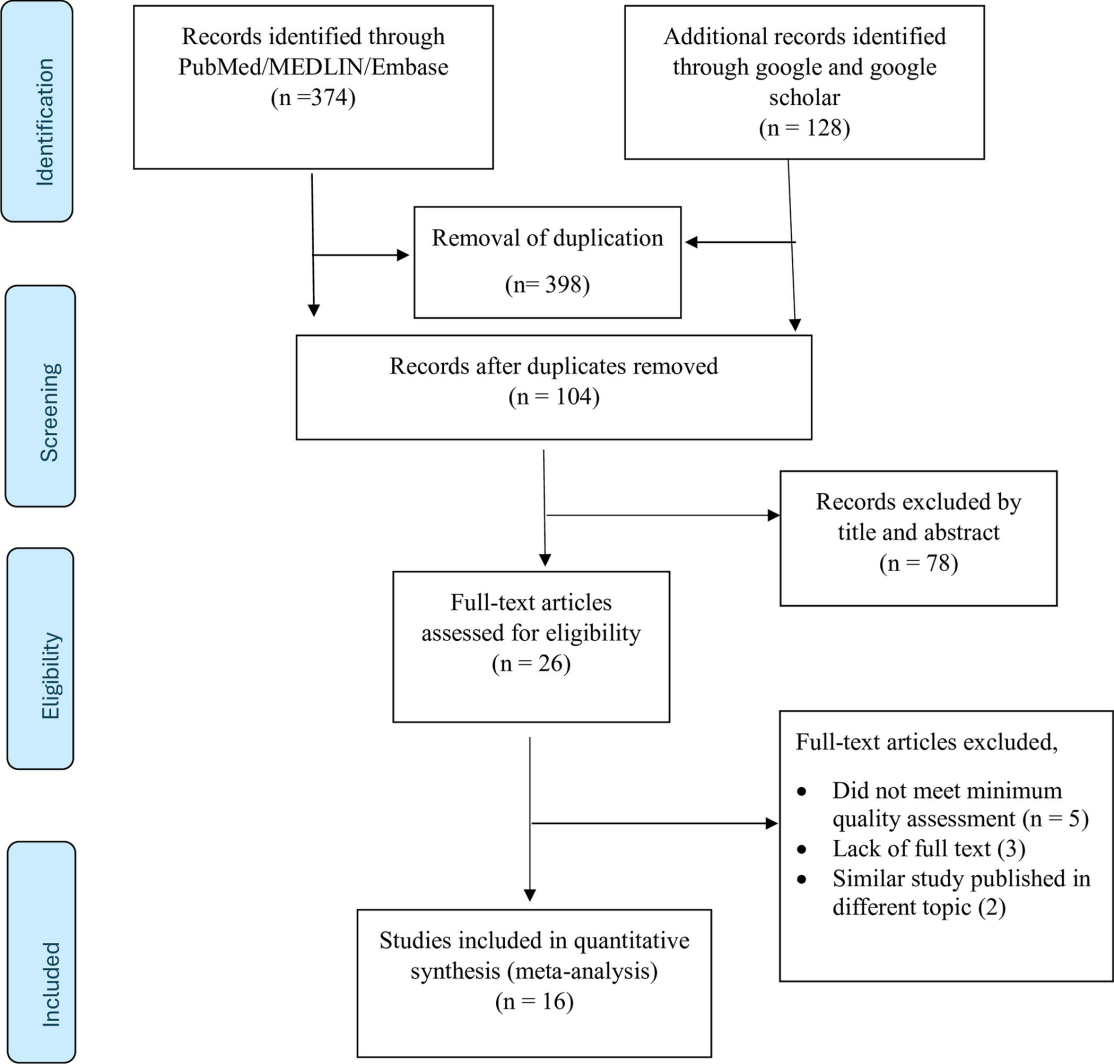
Preferred reporting for systematic review and meta-analysis of CVD in Africa,2024.

A total of 502 initial records were extracted from search engines like PubMed/MEDLINE/Embase, Google, and Google Scholar. From the retrieved records, 398 studies were duplicated, and seventy-eight were excluded after the title and abstract were carefully reviewed based on the eligibility criteria. Full-text article assessment was performed for the rest of the 26 articles, and five, three, and two research articles were excluded from this systematic review and meta-analysis for not meeting the minimum quality assessment [38–42], lack of full-text article access [43–45] and similar studies published using a different topic [46, 47], respectively. Finally, this systematic review and meta-analysis included fifteen articles and one preprint study. The detailed result of all studies identified in the literature search, including those that were excluded from the analyses available in **(Fig 1 and S3 Table)**.

### Study characteristics

In this systematic review and meta-analysis, a total of sixteen cross-sectional studies were included [4, 12, 17, 18, 24–35], with a 21,167-study population. From all studies majority of the included studies were from Ethiopia and Nigeria, respectively. Only one study was included from South Africa and Cameron. All studies assessed congenital CVD. All studies used the Ishihara test to assess the prevalence of CVD, except those conducted in South Africa and Nigeria, which used CVTME and Richmond-HRR tests, respectively. The participants’ ages range from 5 to 60 years. About two-thirds of 11 (68.75%) of the studies, around quarter 4 (25%), and 1 (6.25%) of the studies were conducted in primary and secondary school, university students, and community, respectively **(Table 2).**

**Table 2:** Typical characteristics of cross-sectional studies included in systematic review and meta-analysis of color vision deficiency in Africa, 2024.

Author (year)	Country	Sample size	Source population	Method	Diagnostic criteria	CVD (%)
Gudeta and Asrat et al., (2024) [12]	Ethiopia	864	School Children	Ishihara	> 5 typical red-green defective responses: plates 2 and 21	4.84
Darge et al., (2017) [24]	Ethiopia	378	School Children	-	-	4.2
Mashige et al., (2019) [25]	South Africa	1305	School children	CVTME	> 3 errors: plates 1 and 14	2.2
Dohvoma et al., (2018) [26]	Cameron	303	Higher education	Ishihara	> 3 three typical red-green defective responses: plates 2 and 21	1.7
Wale et al., (2018) [27]	Ethiopia	854	School children	Ishihara	≤ 9 read correctly	4.24
Mitiku et al., (2020) [28]	Ethiopia	4004	Higher education	Ishihara	≤ 9 read correctly	2.85
Ugalahi et al., (2016) [29]	Nigeria	1635	Secondary school	Ishihara	Incorrect response in > 2 plates	2.35
Oduntan et al., (2019) [17]	Nigeria	2326	Primary and secondary school	Richmond-HRR	If any of plates 7–10 were not ticked	2.5
Tabansi et al., (2008) [30]	Nigeria	1300	Primary school	Ishihara	Incorrect response in >2 plates	2.6
Woldeamanuel and Geta, (2018) [18]	Ethiopia	844	School children	Ishihara	> 5 typical red-green defective responses: plates 2 and 21	4.1
Mulusew et al., (2013) [31]	Ethiopia	1040	School children	Ishihara	> 5 typical red-green defective responses: plates 2 and 21	4.2
Eze et al., 2020 [4]	Nigeria	950	Secondary school	Ishihara	Failed to read > 4 letters: plates 1–21	1.2
Mengesha et al., (2021) [32]	Ethiopia	2400	Primary School	Ishihara	≤ 9 read correctly	2.29
Fakorede et al., (2022) [33]	Nigeria	1191	Higher education	Ishihara	≤ 9 read correctly	2.85
Ativie et al., (2017) [34]	Nigeria	1500	Community	Ishihara	≤ 9 read correctly	1.7
Nwobodo et al., (2022) [35]	Nigeria	291	Higher education	Ishihara	Students that failed ≥ 8plates	1.7

CVD; color/colour vision deficiency, CVTME; Color Vision Testing Made Easy, HRR; Hardy-Rand-Rittler

Among the included studies, only six reported on factors associated with CVD. These studies showed that being male [4, 17, 18, 27, 29] and visually impaired [27, 47] were factors associated with CVD in Africa.

### Pooled prevalence of color vision deficiency in Africa

**Fig 2:**
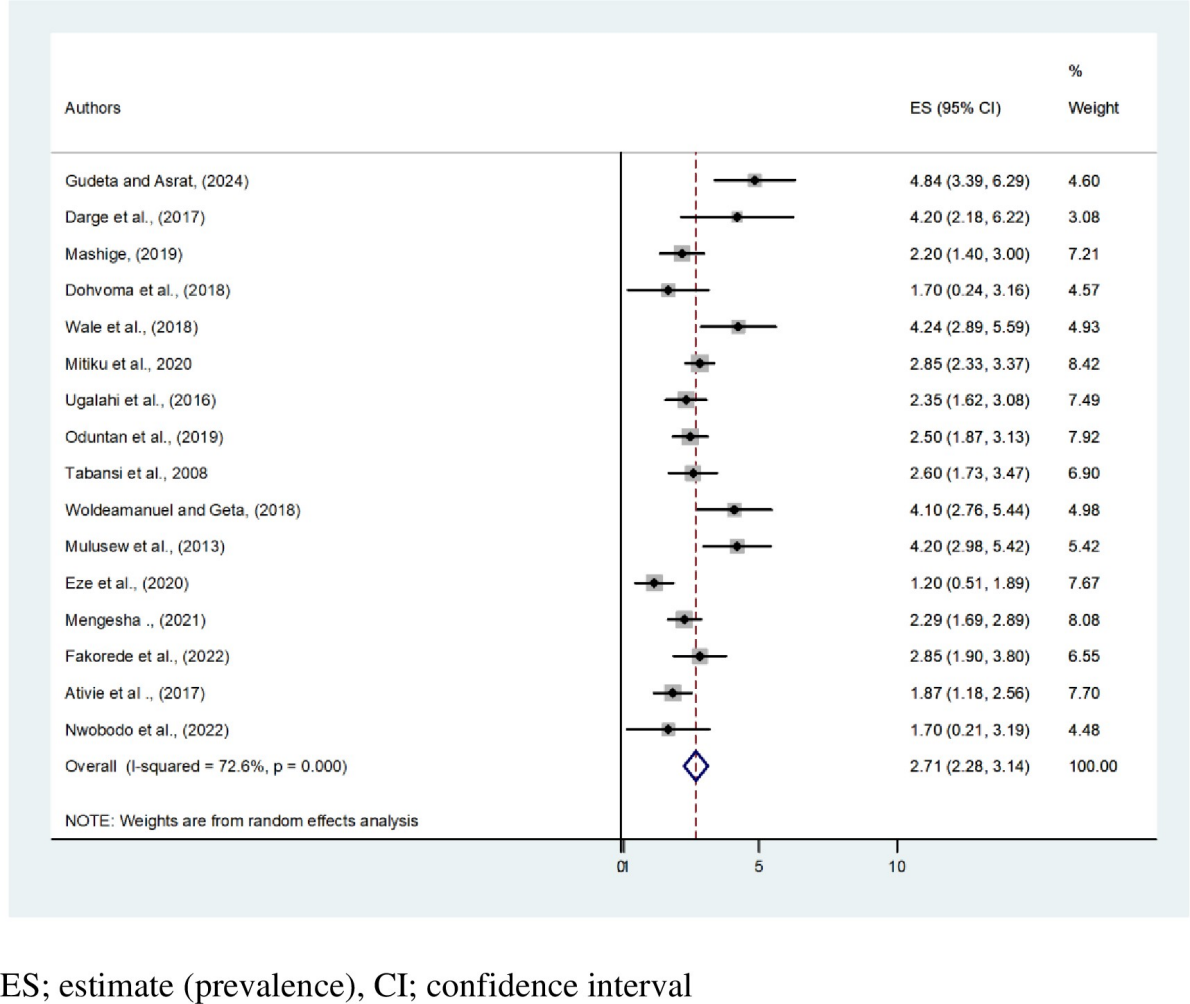
Forest plot shows pooled estimate prevalence of color vision deficiency in Africa.

From all sixteen studies, a total of 21,167 participants were included in this pooled estimate of the prevalence of CVD in Africa. The overall pooled prevalence of CVD in Africa was 2.71 percent (95% CI: 2.28,3.14, I^2^ = 72.6%, P<0.001). The lowest prevalence of CVD (1.20%)(95% CI:0.51,1.89) was recorded in Nigeria among secondary school students [4]. Whereas the higher prevalence of CVD of 4.84% (95% CI:3.39,6.29) was recorded in Ethiopia among primary school children [12] **(Fig 2)**.

### Publication bias

**Fig 3:**
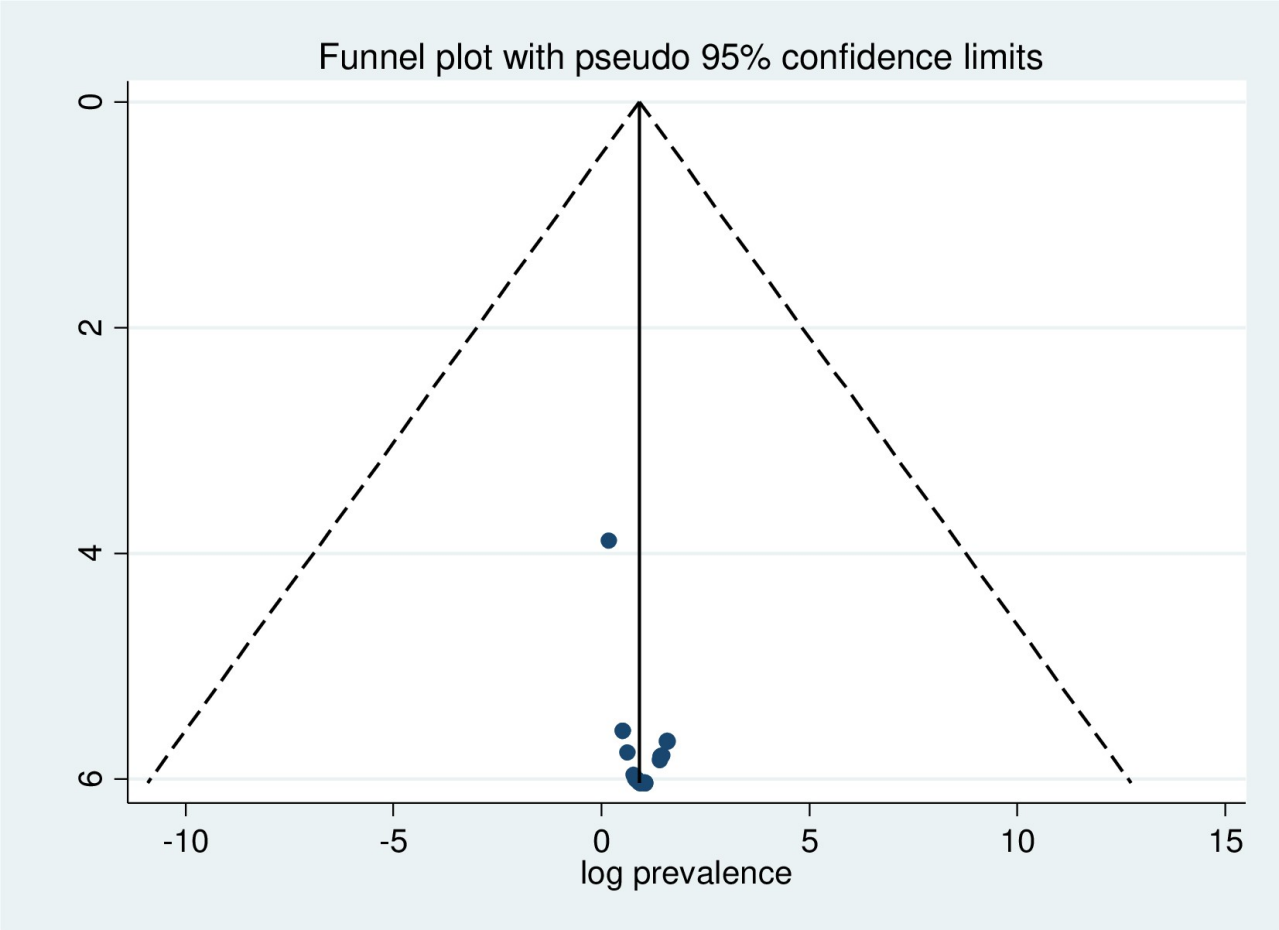
Funnel plot test of the sixteen studies included in the meta-analysis of color vision deficiency in Africa, 2024.

The funnel plot of this systematic review and meta-analysis showed that all the studies were symmetric **(Fig 3),** and the Egger test showed there was no significant publication bias (P-value = 0.067).

### Pooled prevalence of color vision deficiency based on gender in Africa

**Fig 4:**
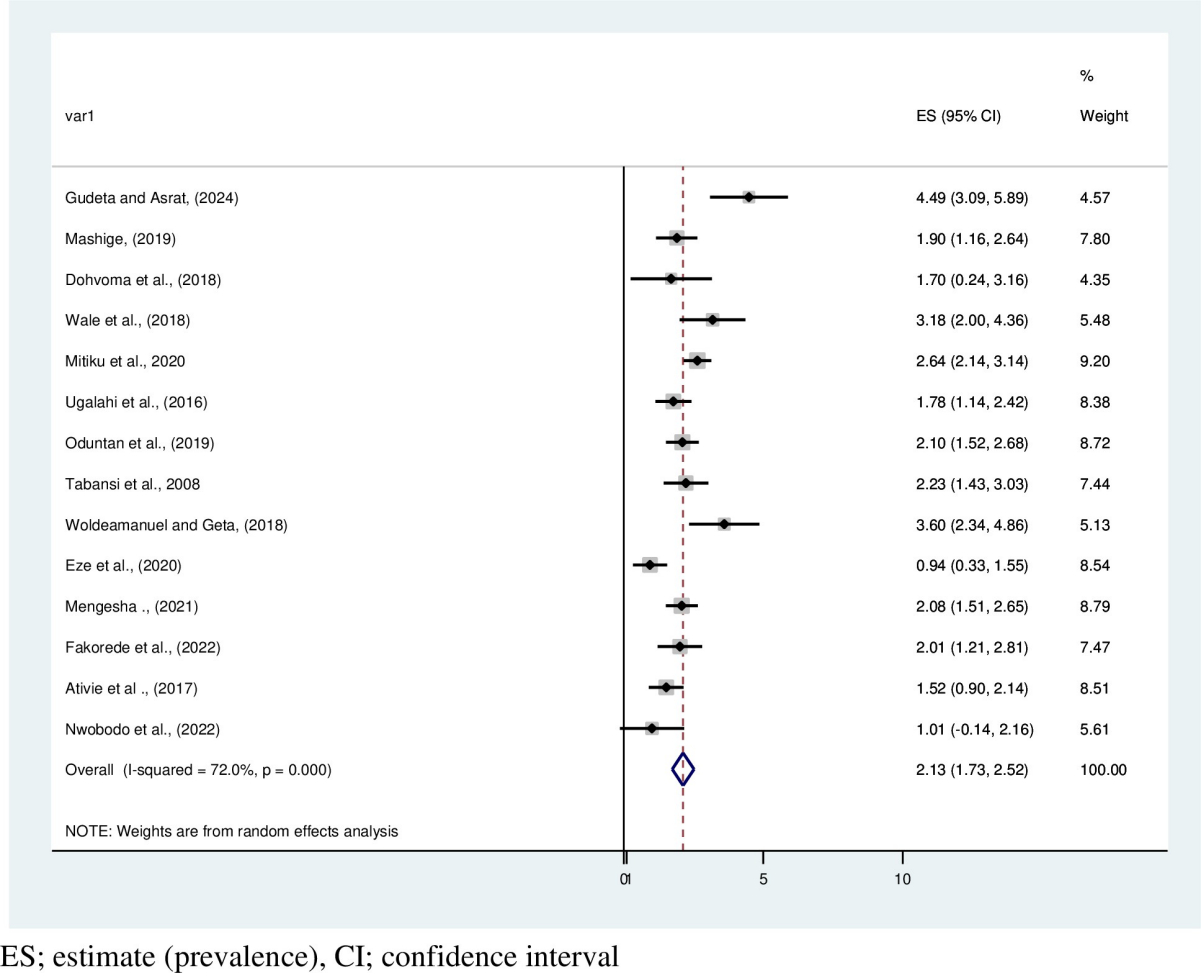
Forest plot on pooled estimate prevalence of color vision deficiency among males in Africa.

**Fig 5:**
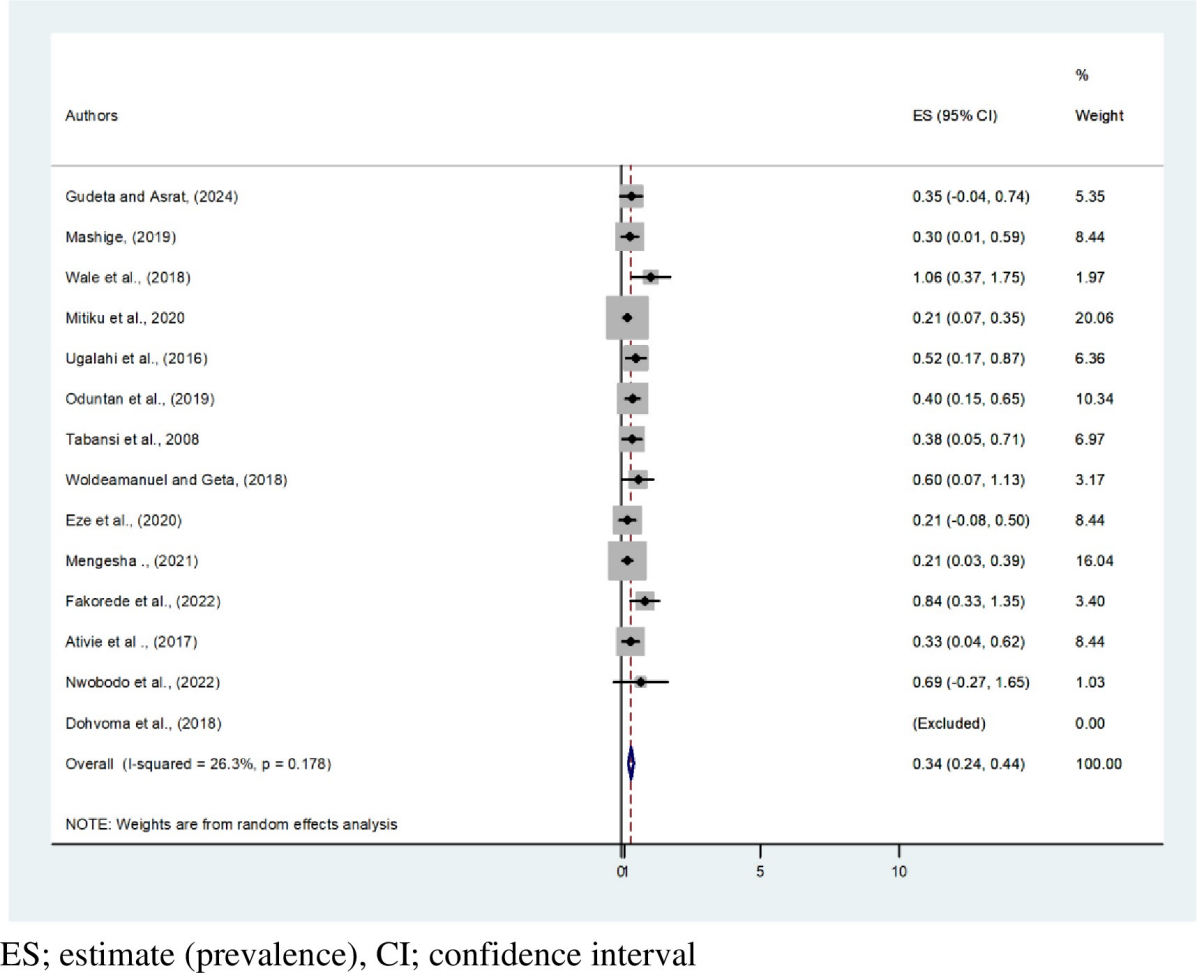
Forest plot on pooled estimate prevalence of color vision deficiency among females in Africa.

This systematic review and meta-analysis showed that the pooled estimated prevalence of CVD among African males and females is 2.13% (95% CI: 1.73, 2.52, I^2^ = 72%, P < 0.001) and 0.34% (95% CI: 0.24,0.44), respectively. The highest prevalence of CVD by gender, 4.49% [12], was recorded among males. Based on this systematic review and meta-analysis, CVD affects 1 in every 35 men and 1 in every 300 females in Africa **(Figs 4 and 5)**. A tolerable heterogeneity level was observed, allowing for a valid comparison among females (I^2^ = 26.3%, P = 0.178).

### Subgroup analysis based on country and source population

**Fig 6:**
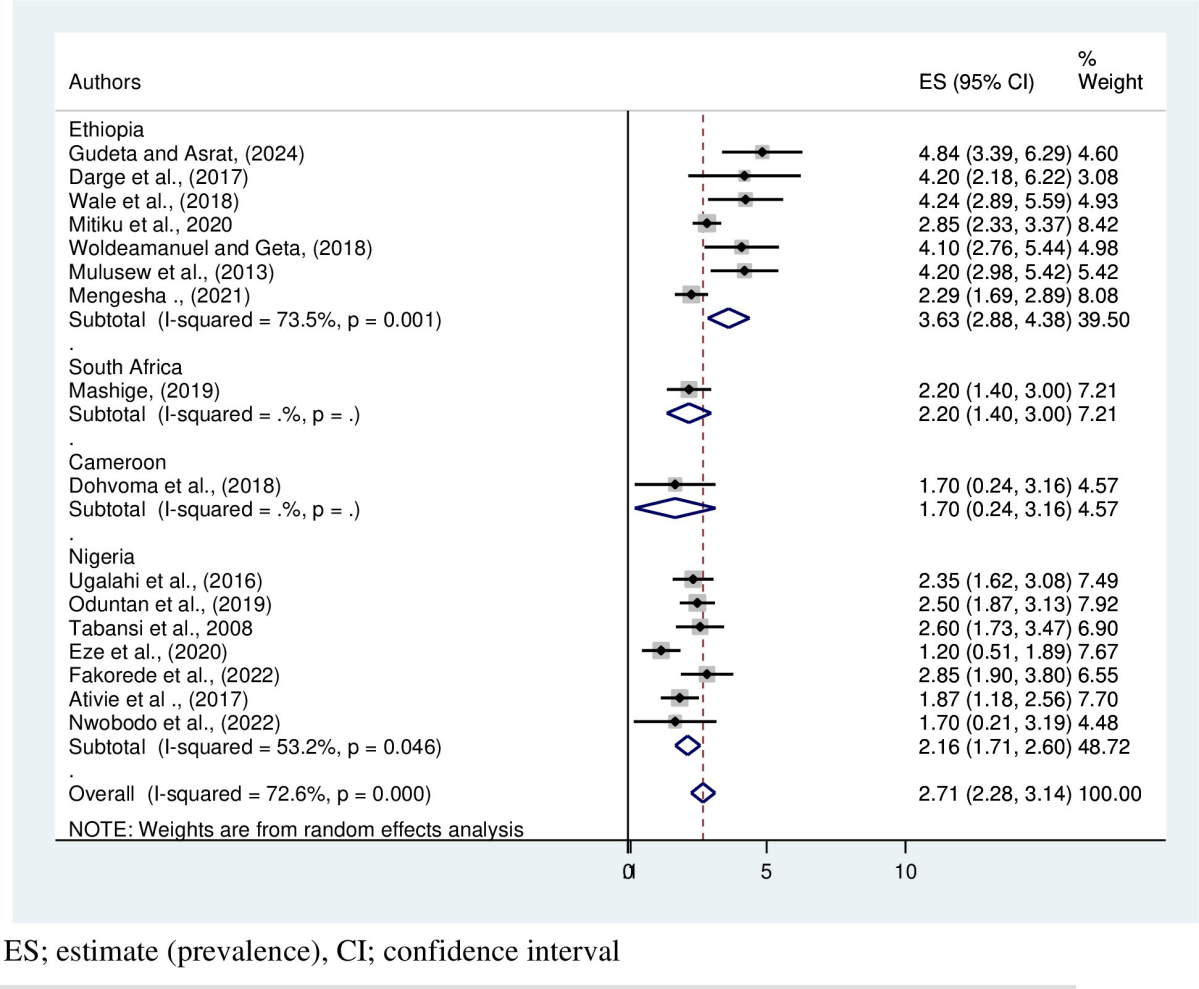
Forest plot on subgroup estimate prevalence of color vision deficiency in Africa based on the country.

The subgroup analysis conducted based on the country showed that the highest pooled prevalence of CVD was recorded in Ethiopia (3.63%, 95% CI:2.88,4.38, I^2^; 73.5, P = 0.001), and the prevalence of CVD ranges from 4.84% (95% CI:3.39,6.29) [12] to 2.29 (95% CI:1.69,2.89) [32] in Ethiopia. Cameroon has the least pooled CVD: 1.70 (95% CI:0.24, 3.16) [26] **(Fig 6)**.

**Fig 7:**
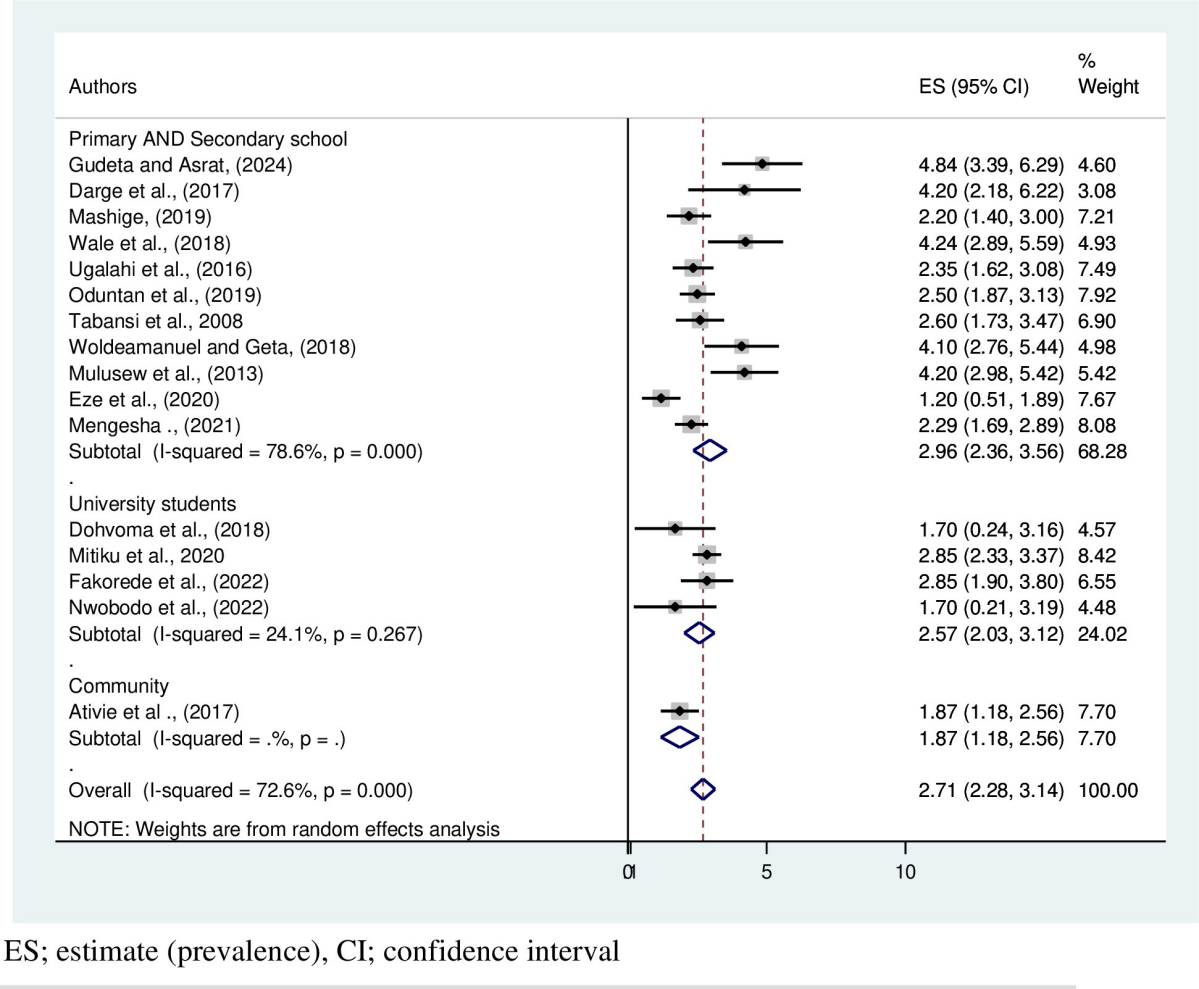
Forest plot on subgroup estimates the prevalence of color vision deficiency in Africa based on the source of population.

In addition, the sub group analysis was conducted based on the source of population, and the highest pooled prevalence of CVD was recorded among primary and secondary school students (2.96%, 95% CI: 2.36, 3.56, I^2^: 78.6%, P = 0.267) **(Fig 7)**.

### Sensitivity analysis

By excluding all 16 articles included step by step, a sensitivity analysis was conducted to test the effect of each study on the pooled prevalence **(Table 3)**.

**Table 3:** Sensitivity analysis of systematic review and meta-analysis of color vision deficiency in Africa, 2024.

Author(year)	Pooled estimate	I ^2^	P value
Gudeta and Asrat et al., (2024) [12]	2.58% (2.18–2.99)	68.5%	<0.001
Darge et al., (2017) [24]	2.66% (2.22–3.09)	73.1%	<0.001
Mashige et al., (2019) [25]	2.75% (2.29–3.21)	74.1%	<0.001
Dohvoma et al., (2018) [26]	2.76% (2.31–3.20)	73.8%	<0.001
Wale et al., (2018) [27]	2.62% (2.19–3.04)	71.0%	<0.001
Mitiku et al., (2020) [28]	2.71% (2.23–3.18)	73.4%	<0.001
Ugalahi et al., (2016) [29]	2.75% (2.28–3.21)	74.3%	<0.001
Oduntan et al., (2019) [17]	2.74% (2.26–3.21)	74.4%	<0.001
Woldeamanuel and Geta, (2018) [18]	2.63% (2.20–3.05)	71.5%	<0.001
Tabansi et al., (2008) [30]	2.72% (2.26–3.18)	74.4%	<0.001
Mulusew et al., (2013) [31]	2.61% (2.19–3.03)	70.3%	<0.001
Eze et al., (2020) [4]	2.81% (2.40–3.21)	64.6%	<0.001
Fakorede et al., (2022) [33]	2.70% (2.25–3.16)	74.2%	<0.001
Ativie et al., (2017) [34]	2.78% (2.33–3.23)	72.5%	<0.001
Nwobodo et al., (2022) [35]	2.76% (2.31–3.12)	73.8%	<0.001
Mengesha et al., (2021) [32]	2.56% (2.28–3.23)	74.1%	<0.001

## Discussion

Studies have shown that the prevalence of CVD in the African population ranges from 1.20% to 4.84% [4, 12]. Therefore, this systematic review and meta-analysis will be crucial for enhancing the overall understanding of CVD in Africa and providing valuable insights to advocate for better management of CVD. In this study, the pooled prevalence of CVD was found to be 2.71% (95% CI: 2.28, 3.14, I^2^ = 72.6%). Furthermore, the prevalence of CVD was 2.13% among African males and 0.34% among African females.

The findings of our current study are consistent with individual studies conducted in South America, which reported a prevalence of 2.36% [48], and a study conducted in Asia, which reported 2.72% [49]. This similarity could be attributed to comparable data collection methods, particularly the use of pseudoisochromatic plates [27, 28, 32].

The findings of the current study show a higher prevalence of color vision deficiency compared to individual reports from Asia: 1% in Bangladesh [50], 1.17% in Saudi Arabia [10], 2.18% in India [51], 2.1% in Nepal [52], and 3.8% in Iran [53]. In North America, an individual report showed a prevalence of 1.4% [54]. Previous single studies had limited sample sizes, particularly among children. In contrast, the current meta-analysis included studies conducted with both children and adults, which may have contributed to the higher prevalence of acquired color vision deficiency [5]. Additionally, ethnic differences among study participants could also accounts for discrepancy [6].

The pooled prevalence of CVD in Africa, as determined by this systematic review and meta-analysis, is lower than the prevalence reported in individual studies from North America, where a 29% prevalence was found among participants with high exposure to both hexane and non-hexane solvents, which was associated with a higher prevalence of acquired color vision defects [7]. In Europe, a single study reported a prevalence of 7.33% [9], and a study from Asia found a prevalence of 6.8% [11], which only included male participants. This might be a possible reason for the higher prevalence, as color vision deficiency is linked to the X linked inheritance pattern and is more prevalent in men [2].

In a systematic review and meta-analysis, the prevalence of CVD was found to be 2.13% among African males, and 0.34% among African females. In Iran, a systematic review and meta-analysis reported a prevalence of 4.7% among males and 0.7% among females [53]. The difference between the two populations could be attributed to ethnic variation, possibly related to variance in color vision among different ethnic groups [6].

The highest pooled prevalence of CVD was recorded in Ethiopia at 3.63%, followed by South Africa at 2.20%, and Nigeria at 2.16%, with the lowest prevalence in Cameroon at 1.70% [26]. A higher percentage of male participants were enrolled in Ethiopia studies compared to Cameroon, where there was a comparable number of male and female participants. The difference in prevalence may be attributed to the fact that CVD primarily affects male [53] and ethnic variation between the two population [6]. These factors should be considered in future studies.

The highest pooled prevalence of CVD was found among primary and secondary school students, followed by studies conducted among university students and community-based studies. More participants were involved in studies conducted among primary and secondary school students, with a higher percentage of male participants compared to studies conducted among university students and community-based studies. Since CVD follows an X-linked pattern [29], this could explain the higher prevalence among primary and secondary school students.

In a systematic review and meta-analysis, being male [4, 17, 18, 27, 29] and visually impaired [27, 47] were frequently reported as risk factors for CVD in Africa. Males had a higher prevalence of CVD than females due to genetic predisposition [29]. Another significant factor is visual impairment, as those who are visually impaired are more likely to acquired CVD [47].

Color vision deficiency poses a significant challenge in many occupational activities. A diagnosis of CVD can have a negative psychological impact on mental well-being [55] and can adversely affect job prospects [56], often leading individuals to invest significant time and resources, both financial and mental, toward an unattainable goal [14, 57]. Its high prevalence among school-age children and university students can negatively affect their academic performance [58]. Raising public awareness through mainstream media and social media is crucial. Parents should be informed about color vision deficiency in their children, enabling early diagnosis and management to support their kids [59].

Regular early screening and counseling programs at higher education centers are essential to minimize the impact of CVD on African students. It is critical to ensure that individuals and children receive proper care without it interfering with their future careers and education. Research should be conducted on the occupational impact of CVD in Africa, advocating for changes in recruitment process based on the type and severity of color vision loss. Additionally, critical color-related tasks in the work environment should be considered for future planning in Africa.

### Limitations

High heterogenicity was observed in the estimate of the pooled prevalence of CVD and in sub group estimate based on country and source population. This variation may be attributed to the difference in the percentage of sex and ethnicity across the studies included in the analysis, which could influence the prevalence. Different studies used different pass/fail criteria on the Ishihara test and this could impact the measured prevalence of CVD. Additionally, the inability to access three full text articles was a limitation of this systematic review and meta-analysis.

### Conclusion

The prevalence of color vision deficiency in Africa was found to be 2.71%, with 2.13% among African males and 0.34% among African females. It is critical to increase public awareness and establish proper screening programs for school children and university students.

## Supporting information

S1 Checklist PRISMA 2020 checklist. (DOCX)

S1 Table Data extraction sheet. (DOCX)

S2 Table Quality assessment/risk of bias. (DOCX)

S3 Table (DOCX)

S1 Dataset (XLSX)
