# Supplementary material for: Prevalence of color vision deficiency in Africa: Systematic review and meta-analysis
Source: PLoS One. 2024 Dec 4;19(12):e0313819. doi: 10.1371/journal.pone.0313819 (PMC11616826; doi:10.1371/journal.pone.0313819)
Supplement: S1 Table — (DOCX) [file pone.0313819.s002.docx]

**Table S1: Data extraction sheet**

| Each studies data was extracted by 3 review authors (MMT, AGJ, and FDS) independently. The data was extracted from June, 03, 2024 to July 1, 2024 | | | | | | | | | | | | | |
| --- | --- | --- | --- | --- | --- | --- | --- | --- | --- | --- | --- | --- | --- |
| Author (year) | Year of study | Country | Sample size | Study design | Source population | Age interval (years) | Method of assessment | Type of CVD assessed | Diagnostic criteria | CVD (%) | Male (%) | Female (%) | eligibility |
| Gudeta and Asrat et al., (2024) [12] | 2015 | Ethiopia | 864 | Cross sectional | School Children | 9-16 | Ishihara | CVD | > 5 typical red-green defective responses: plates 2 and 21 | 4.84 | 4.49% | 0.35% | Eligible |
| Darge et al., (2017) [24] | 2015 | Ethiopia | 378 | Cross sectional | School Children | 5-16 | - | CVD | - | 4.2 | - | - | Eligible |
| Mashige et al., (2019) [25] | 2017 | South Africa | 1305 | Cross sectional | School children | 7-17 | CVTME | CVD | > 3 errors: plates 1 and 14 | 2.2 | 1.90% | 0.30% | Eligible |
| Dohvoma et al., (2018) [26] | 2015 | Cameron | 303 | Cross sectional | Higher education | N.R | Ishihara | CVD | > 3 three typical red-green defective responses: plates 2 and 21 | 1.7 | 1.70% | 0% | Eligible |
| Wale et al., (2018) [27] | 2016 | Ethiopia | 854 | Cross sectional | School children | 8-18 | Ishihara | Color blindness | ≤ 9 read correctly | 4.24 | 3.18% | 1.06% | Eligible |
| Mitiku et al., (2020)[28] | N.R | Ethiopia | 4004 | Cross sectional | Higher education | 18-47 | Ishihara | CVD | ≤ 9 read correctly | 2.85 | 2.64% | 0.21% | Eligible |
| Ugalahi et al., (2016) [29] | N.R | Nigeria | 1635 | Cross sectional | Secondary school | N.R | Ishihara | CVD | Incorrect response in > 2 plates | 2.35 | 1.78% | 0.52% | Eligible |
| Oduntan et al., (2019) [17] | N.R | Nigeria | 2326 | Cross sectional | Primary and secondary school | 7-22 | Richmond-HRR | CVD | If any of plates 7-10 were not ticked | 2.5 | 2.10% | 0.40% | Eligible |
| Tabansi et al., (2008) [30] | N.R | Nigeria | 1300 | Cross sectional | Primary school | N.R | Ishihara | CVD | Incorrect response in >2 plates | 2.6 | 0.40% | 2.20% | Eligible |
|  |  |  |  |  |  |  |  |  |  |  |  |  |  |
| Woldeamanuel and Geta, (2018) [18] | N.R | Ethiopia | 844 | Cross sectional | School children | 7-18 | Ishihara | CVD | > 5 typical red-green defective responses: plates 2 and 21 | 4.1 | 3.60% | 0.60% | Eligible |
| Mulusew et al., (2013)[31] | 2009 | Ethiopia | 1040 | Cross sectional | School children | N.R | Ishihara | CVD | > 5 typical red-green defective responses: plates 2 and 21 | 4.2 | - | - | Eligible |
| Eze et al., 2020 [4] | N.R | Nigeria | 950 | Cross sectional | Secondary school | 10-20 | Ishihara | CVD | Failed to read > 4 letters: plates 1-21 | 1.2 | 0.94% | 0.21% | Eligible |
| Mengesha et al., (2021)[32] | N.R | Ethiopia | 2400 | Cross sectional | Primary School | 12-16 | Ishihara | CVD | ≤ 9 read correctly | 2.29 | 2.08% | 0.21% | Eligible |
| Fakorede et al., (2022) [33] | N.R | Nigeria | 1191 | Cross sectional | Higher education | 18-39 | Ishihara | CVD | ≤ 9 read correctly | 2.85 | 2.01% | 0.84% | Eligible |
| Ativie et al., (2017)[34] | N.R | Nigeria | 1500 | Cross sectional | Community | 10-60 | Ishihara | CVD | ≤ 9 read correctly | 1.87 | 1.52% | 0.33% | Eligible |
| Nwobodo et al., (2022)[35] | N.R | Nigeria | 291 | Cross sectional | Higher education | N.R | Ishihara | CVD | Students that failed ≥ 8plates | 1.7 | 1.01% | 0.69% | Eligible |
