# Supplementary material for: Prevalence of color vision deficiency in Africa: Systematic review and meta-analysis
Source: PLoS One. 2024 Dec 4;19(12):e0313819. doi: 10.1371/journal.pone.0313819 (PMC11616826; doi:10.1371/journal.pone.0313819)
Supplement: S2 Table — (DOCX) [file pone.0313819.s003.docx]

S2 Table: Quality assessment/risk of bias

| Authors | Q1, Sample frame appropriate to address the target population | Q2, Study participants sampled in an appropriate way | Q3, Sample size adequate | Q4, Study subjects and the setting described in detail | Q5, Data analysis conducted with sufficient coverage of identified sample | Q6, Valid methods used for the identification of the condition | Q7, Condition measured in a standard, reliable way for all participants | Q8, there appropriate statistical analysis | Q9, the response rate adequate, and if not, was the low response rate managed appropriately | score (9) | Risk of bias |
| --- | --- | --- | --- | --- | --- | --- | --- | --- | --- | --- | --- |
| Gudeta and Asrat et al., [12] | Yes | No | Yes | Yes | Yes | Yes | Yes | No | Yes | 6 | Low |
| Darge et al., [24] | U/c | Yes | Yes | Yes | U/c | U/c | U/c | Yes | Yes | 5 | Low |
| Mashige et al., [25] | Yes | Yes | Yes | Yes | Yes | Yes | Yes | Yes | Yes | 9 | Low |
| Dohvoma et al., [26] | No | No | Yes | Yes | Yes | Yes | Yes | Yes | Yes | 7 | Low |
| Wale et al., [27] | Yes | Yes | Yes | Yes | Yes | U/c | Yes | No | Yes | 7 | Low |
| Mitiku et al, [28] | Yes | U/c | U/c | Yes | Yes | Yes | U/c | Yes | Yes | 6 | Low |
| Ugalahi et al., [29] | Yes | Yes | Yes | Yes | Yes | Yes | U/c | No | Yes | 7 | Low |
| Oduntan et al.,[17] | Yes | Yes | Yes | Yes | Yes | Yes | Yes | Yes | Yes | 9 | Low |
| Tabansi et al., [30] | Yes | No | Yes | Yes | Yes | Yes | Yes | No | Yes | 7 | Low |
| Woldeamanuel et al., [18] | Yes | Yes | Yes | Yes | Yes | Yes | Yes | No | Yes | 8 | Low |
| Mulusew et al., [31] | Yes | No | Yes | Yes | Yes | Yes | U/c | Yes | Yes | 7 | Low |
| Eze et al., [4] | Yes | Yes | U/c | Yes | U/c | Yes | Yes | Yes | U/c | 6 | Low |
| Mengesha et al., [32] | No | Yes | Yes | Yes | Yes | Yes | U/c | Yes | Yes | 7 | Low |
| Fakorede et al, [33] | Yes | U/c | Yes | Yes | Yes | U/c | Yes | U/c | Yes | 6 | Low |
| Ativie et al., [34] | Yes | Yes | Yes | Yes | Yes | Yes | U/c | No | Yes | 7 | Low |
| Nwobodo et al., [35] | Yes | Yes | No | Yes | Yes | Yes | U/c | No | Yes | 6 | Low |

Q1; was the sample frame appropriate to address the target population? Q2= were study participants sampled in an appropriate way? Q3; was the sample size adequate? Q4; were the study subjects and the setting described in detail? Q5; was the data analysis conducted with sufficient coverage of the identified sample? Q6; were valid methods used for the identification of the condition? Q7; was the condition measured in a standard, reliable way for all participants? Q8; was there appropriate statistical analysis? Q9; was the response rate adequate, and if not, was the low response rate managed appropriately? U/c; unclear
