# Supplementary material for: Prevalence of color vision deficiency in Africa: Systematic review and meta-analysis
Source: PLoS One. 2024 Dec 4;19(12):e0313819. doi: 10.1371/journal.pone.0313819 (PMC11616826; doi:10.1371/journal.pone.0313819)
Supplement: S3 Table — (DOCX) [file pone.0313819.s004.docx]

S3 table:

| Studies identified in the literature search, including those that were excluded from the analyses. | | | Number of Articles | |
| --- | --- | --- | --- | --- |
| 1. | Initially identified studies | | 502 | |
| 2. | Duplicated studies and removed | | 398 | |
| 3. | excluded after the title and abstract were carefully reviewed based on the eligibility criteria | | 78 | |
| Tile and reference of excluded studies after title and abstract review | | Reason for exclusion | | |
| 3.1 | **Title:** Prevalence of Color Blindness in Iranian Students: A Meta-analysis  Citation: Rezaei L, Hawasi E, Salari N, Mohammadi M. Prevalence of Color Blindness in Iranian Students: A Meta-analysis. Journal of Ophthalmic & Vision Research. 2022 Jul;17(3):413. | | The study is meta-analysis and was conducted among Iranian students. | |
| 3.2 | **Title:** Prevalence of Color Vision Anomalies among Dental Professionals  **Citation**: Ngente Z, Jadav B, Aafaque S, Patil A, Govindarajulu R, Muthusamy P, Babu JS, Swarnalatha C, Nayyar AS. Prevalence of color vision anomalies among dental professionals. Middle East African Journal of Ophthalmology. 2021 Jul 1;28(3):169-73. | | The study was not conducted in Africa (India) | |
| 3.3 | **Title:** Colour-emotion associations in individuals with red-green colour blindness  **Citation**: Jonauskaite D, Camenzind L, Parraga CA, Diouf CN, Ducommun MM, Müller L, Norberg M, Mohr C. Colour-emotion associations in individuals with red-green colour blindness. PeerJ. 2021 Apr 7;9:e11180. | | The study was experimental and out of the study scope | |
| 3.4 | **Title:** Prevalence of color vision deficiency among school-going boys in South India  **Citation:** Krishnamurthy SS, Rangavittal S, Chandrasekar A, Narayanan A. Prevalence of color vision deficiency among school-going boys in South India. Indian Journal of Ophthalmology. 2021 Aug 1;69(8). | | The study was not conducted in Africa (India) | |
| 3.5 | **Title:** Prevalence of color vision deficiency among arc welders  **Citation:** Heydarian S, Mahjoob M, Gholami A, Veysi S, Mohammadi M. Prevalence of color vision deficiency among arc welders. Journal of optometry. 2017 Apr 1;10(2):130-4. | | The study was not conducted in Africa (Iran) and its comparative study | |
| 3.6 | **Title:** Prevalence of color vision deficiency in medical students at a Saudi University  **Citation:** Alamoudi NB, AlShammari RZ, AlOmar RS, AlShamlan NA, Alqahtani AA, AlAmer NA. Prevalence of color vision deficiency in medical students at a Saudi University. Journal of Family and Community Medicine. 2021 Sep 1;28(3):196-201. | | The study was not conducted in Africa (Saudi Arabia) | |
| 3.7 | **Title:** Association Between Color Vision Deficiency and Myopia in Chinese Children Over a Five-Year Period  **Citation:** Gan J, Li SM, Atchison DA, Kang MT, Wei S, He X, Bai W, Li H, Kang Y, Cai Z, Li L. Association between color vision deficiency and myopia in Chinese children over a five-year period. Investigative Ophthalmology & Visual Science. 2022 Feb 1;63(2):2-. | | The study was not conducted in Africa (China) | |
| 3.8 | **Title:** Dalton's pseudo-isochromatic plates and congenital colour vision deficiency  **Citation:** Narayanan A, Venkadesan M, Krishnamurthy SS, Hussaindeen JR, Ramani KK. Dalton's pseudo‐isochromatic plates and congenital colour vision deficiency. Clinical and Experimental Optometry. 2020 Nov 1;103(6):853-7. | | The study was not conducted in Africa and the outcome of interests is not measured | |
| 3.9 | **Title:** Prevalence and Determinants of Color Vision Defects among Preparatory University Students at Makkah, Saudi Arabia  **Citation:** Khairoalsindi OA, Almasoudi BM, Bamahfouz AY, Alghamdi AA, Siddiqui MI. Prevalence and determinants of color vision defects among preparatory university students at Makkah, Saudi Arabia. Middle East African Journal of Ophthalmology. 2019 Jul 1;26(3):133-7. | | The study was not conducted in Africa (Saudi Arabia) | |
| 3.10 | **Title:** Prevalence of color vision deficiency among Chinese college students and their quality of life  **Citation:** Gao JG, Tian M. Prevalence of color vision deficiency among Chinese college students and their quality of life. International Journal of Ophthalmology. 2023;16(9):1542. | | The study was not conducted in Africa China) | |
| 3.11 | **Title:** The prevalence of color vision deficiency in the northeast of Iran  Citation: Hashemi H, Khabazkhoob M, Pakzad R, Yekta A, Heravian J, Nabovati P, Ostadimoghaddam H. The prevalence of color vision deficiency in the northeast of Iran. Journal of current ophthalmology. 2019 Mar 1;31(1):80-5. | | The study was not conducted in Africa (Iran) | |
| 3.12 | **Title:** Prevalence of Color Blindness in Undergraduates of Kathmandu University  **Citation:** Jha RK, Khadka S, Gautam Y, Bade M, Jha MK, Nepal O. Prevalence of Color Blindness in Undergraduates of Kathmandu University. JNMA: Journal of the Nepal Medical Association. 2018 Nov;56(214):900. | | The study was not conducted in Africa (Nepal) | |
| 3.13 | **Title:** Quantitative and objective diagnosis of color vision deficiencies based on steady-state visual evoked potentials  **Citation:** Zheng X, Xu G, Wang Y, Du C, Liang R, Zhang K, Jia Y, Du Y, Zhang S. Quantitative and objective diagnosis of color vision deficiencies based on steady-state visual evoked potentials. International Ophthalmology. 2021 Feb;41:587-98. | | The study was not conducted in Africa and its color vision testing method validation | |
| 3.14 | **Title:** Prevalence of colour vision deficiency in the Republic of Ireland schoolchildren and associated socio-demographic factors  **Citation:** Harrington S, Davison PA, O'dwyer V. Prevalence of colour vision deficiency in the Republic of Ireland schoolchildren and associated socio-demographic factors. Clinical and Experimental Optometry. 2021 Jan 2;104(1):48-55. | | The study was not conducted in Africa (Ireland) | |
| 3.15 | **Title:** Prevalence of color vision deficiency in an adult population in South Korea  **Citation:** Kim H, Ng JS. Prevalence of color vision deficiency in an adult population in South Korea. Optometry and Vision Science. 2019 Nov 1;96(11):866-73. | | The study was not conducted in Africa (South Korea) | |
| 3.16 | **Title:** Aviation-relevent epidemiology of color vision deficiency  **Citation:** Delpero WT, O’Neill H, Casson E, Hovis J. Aviation-relevent epidemiology of color vision deficiency. Aviation, space, and environmental medicine. 2005 Feb 1;76(2):127-33. | | This study was a Review paper | |
| 3.17 | **Title:** Colour vision deficiency and sputum colour charts in COPD patients: an exploratory mixed-method study  **Citation:** Channa S, Gale N, Lai E, Hall L, Quinn M, Turner AM. Colour vision deficiency and sputum colour charts in COPD patients: an exploratory mixed-method study. NPJ Primary Care Respiratory Medicine. 2021 Mar 4;31(1):13. | | Was conducted to asses impact of CVD on COPD among patients with CVD | |
| 3.18 | **Title:** Color vision deficiency and functional disorders among Israeli male adolescents between 2007 and 2013  **Citation:** Berger A, Findler M, Maymon D, Korach T, Yativ OF, Gronovich Y, Hassidim A. Color vision deficiency and functional disorders among Israeli male adolescents between 2007 and 2013. Journal of Child Neurology. 2016 Sep;31(10):1245-9. | | The study was not conducted in Africa (Israel) | |
| 3.19 | **Title:** Color vision deficiency in Zahedan, Iran: lower than expected.  **Citation:** Momeni-Moghaddam H, Ng JS, Robabi H, Yaghubi F. Color vision deficiency in Zahedan, Iran: lower than expected. Optometry and Vision Science. 2014 Nov 1;91(11):1372-6. | | The study was not conducted in Africa (Iran) | |
| 3.20 | **Title:** Prevalence and gene frequency of color vision impairments among children of six populations from North Indian region.  **Citation:** Fareed M, Anwar MA, Afzal M. Prevalence and gene frequency of color vision impairments among children of six populations from North Indian region. Genes & Diseases. 2015 Jun 1;2(2):211-8. | | The study was not conducted in Africa (Indian) | |
| 3.21 | **Title:** Prevalence of red-green color vision defects among Muslim males and females of Manipur, India.  **Citation:** Ahsana SH, Hussain R, Fareed M, Afzal M. Prevalence of red-green color vision defects among Muslim males and females of Manipur, India. Iranian journal of public health. 2013;42(1):16. | | The study was not conducted in Africa (Indian) | |
| 3.22 | **Title:** The prevalence of red-green color vision deficiency and its related factors in an elderly population above 60 years of age.  **Citation:** Hashemi H, Shahidi A, Hashemi A, Jamali A, Mortazavi A, Khabazkhoob M. The prevalence of red-green color vision deficiency and its related factors in an elderly population above 60 years of age. International Journal of Ophthalmology. 2023;16(9):1535. | | The study was not conducted in Africa (Iran) | |
| 3.23 | **Title:** Color vision deficiency in a middle-aged population: the Shahroud Eye Study.  **Citation:** Jafarzadehpur E, Hashemi H, Emamian MH, Khabazkhoob M, Mehravaran S, Shariati M, Fotouhi A. Color vision deficiency in a middle-aged population: the Shahroud Eye Study. International ophthalmology. 2014 Oct;34:1067-74. | | The study was not conducted in Africa (Iran) | |
| 3.24 | **Title:** Prevalence of Congenital Color Vision Deficiency in Southern Taiwan and Detection of Female Carriers by Visual Pigment Gene Analysis  **Citation:** Kuo HK, Tsao ST, Wu PC. Prevalence of Congenital Color Vision Deficiency in Southern Taiwan and Detection of Female Carriers by Visual Pigment Gene Analysis. International Journal of Molecular Sciences. 2023 Oct 17;24(20):15247. | | The study was not conducted in Africa (Taiwan) | |
| 3.25 | **Title:** Color-discrimination threshold determination using pseudoisochromatic test plates.  **Citation:** Jurasevska K, Ozolinsh M, Fomins S, Gutmane A, Zutere B, Pausus A, Karitans V. Color-discrimination threshold determination using pseudoisochromatic test plates. Frontiers in Psychology. 2014 Nov 27;5:1376. | | The study does not contain outcome of interest. (it assesses test performance, properties, of CVD assessment techniques) | |
| 3.26 | **Title:** Prevalence of colour vision deficiency among medical students and health personnel.  **Citation:** Balasundaram R, Reddy SC. Prevalence of colour vision deficiency among medical students and health personnel. Malaysian family physician: the official journal of the Academy of Family Physicians of Malaysia. 2006;1(2-3):52**.** | | The study was not conducted in Africa (Malysia) | |
| 3.27 | **Title:** Prevalence of color vision deficiency and its correlation with amblyopia and refractive errors among primary school children.  **Citation:** Rajavi Z, Sabbaghi H, Baghini AS, Yaseri M, Sheibani K, Norouzi G. Prevalence of color vision deficiency and its correlation with amblyopia and refractive errors among primary school children. Journal of ophthalmic & vision research. 2015 Apr;10(2):130. | | The study was not conducted in Africa (Iran) | |
| 3.28 | **Title:** Color vision deficiency in preschool children: the multi-ethnic pediatric eye disease study.  Citation: Xie JZ, Tarczy-Hornoch K, Lin J, Cotter SA, Torres M, Varma R, Multi-Ethnic Pediatric Eye Disease Study Group. Color vision deficiency in preschool children: the multi-ethnic pediatric eye disease study. Ophthalmology. 2014 Jul 1;121(7):1469-74. | | The study was not conducted in Africa (California, USA) | |
| 3.29 | **Title:** Ocular abnormalities in beta thalassemia patients: prevalence, impact, and management strategies.  **Citation:** Heydarian S, Jafari R, Dailami KN, Hashemi H, Jafarzadehpour E, Heirani M, Yekta A, Mahjoob M, Khabazkhoob M. Ocular abnormalities in beta thalassemia patients: prevalence, impact, and management strategies. International Ophthalmology. 2020 Feb;40:511-27. | | The study was review of Articles | |
| 3.30 | **Title:** Prevalence and awareness levels of color blindness among students of faculty of dentistry and dental prosthesis technology program.  **Citation:** Ataol AS, Ergun G. Prevalence and awareness levels of color blindness among students of faculty of dentistry and dental prosthesis technology program. European Oral Research. 2022 Sep 1;56(3):149-57. | | The study was not conducted in Africa (turkey) | |
| 3.31 | **Title:** Color vision defects in school going children.  **Citation:** Shrestha RK, Joshi MR, Shakya S, Ghising R. Color vision defects in school going children. Journal of Nepal Medical Association. 2010;50(180). | | The study was not conducted in Africa (Nepal) | |
| 3.32 | **Title:** Incidence of colour blindness in Indians.  **Citation:** Mehra KS. Incidence of colour blindness in Indians. The British Journal of Ophthalmology. 1963 Aug;47(8):485. | | The study was not conducted in Africa (India) | |
| 3.33 | **Title:** Ocular abnormalities in multi-transfused beta-thalassemia patients.  **Citation:** Jafari R, Heydarian S, Karami H, Shektaei MM, Dailami KN, Amiri AA, Rezaee MR, Far AA. Ocular abnormalities in multi-transfused beta-thalassemia patients. Indian Journal of Ophthalmology. 2015 Sep 1;63(9):710-5. | | The study was not conducted in Africa (Iran) and its experimental study | |
| 3.34 | **Title:** Tilted disc syndrome and colour vision.  **Citation:** Vuori ML, Mäntyjärvi M. Tilted disc syndrome and colour vision. Acta Ophthalmologica Scandinavica. 2007 Sep;85(6):648-52. | | The study was not conducted in Africa (Finland) | |
| 3.35 | **Title:** A comparison of red-green color vision deficiency between medical and non-medical students in Pakistan.  **Citation:** Siddiqui QA, Shaikh SA, Qureshi TZ, Subhan MM. A comparison of red-green color vision deficiency between medical and non-medical students in Pakistan. Saudi Med J. 2010 Aug 1;31(8):895-9. | | The study was not conducted in Africa (Pakistan) | |
| 3.36 | **Title:** Protan colour vision deficiency and road accidents.  **Citation:** Cole BL. Protan colour vision deficiency and road accidents. Clinical and experimental optometry. 2002 Jul;85(4):246-53. | | The study was not conducted in Africa (Australia) and the study did’t assessed the prevalence of CVD | |
| 3.37 | **Title:** Acquired colour vision defects in glaucoma—their detection and clinical significance.  **Citation:** Pacheco-Cutillas M, Edgar DF, Sahraie A. Acquired colour vision defects in glaucoma—their detection and clinical significance. British Journal of Ophthalmology. 1999 Dec 1;83(12):1396-402. | | The study was not conducted in Africa (London) and the study did’t assessed the prevalence of CVD | |
| 3.38 | **Title:** The incidence of color blindness among some school children of Pokhara, Western Nepal.  **Citation:** Niroula DR, Saha CG. The incidence of color blindness among some school children of Pokhara, Western Nepal. Nepal Med Coll J. 2010 Mar 1;12(1):48-50. | | The study was not conducted in Africa (Nepal) | |
| 3.39 | **Title:** Burden of ocular morbidities and color blindness among school-attending children in a foothill town of Uttarakhand State.  **Citation:** Mittal SK, Mittal S, Saraswat NK, Kishore S, Agrawal A, Singh A, Samanta R, Bahurupi YA. Burden of ocular morbidities and color blindness among school-attending children in a foothill town of Uttarakhand State. Indian Journal of Ophthalmology. 2022 Jan 1;70(1):249-55. | | The study was not conducted in Africa (India) | |
| 3.40 | **Title: Eye disorders in old people.**  **Citation:** Khalaj M, Barikani A, Ghasemi H. Eye disorders in old people. Global Journal of Health Science. 2012 Nov 6;5(1):79. | | The study was not conducted in Africa (Iran) | |
| 3.41 | **Title:** A large population study reveals a novel association between congenital color vision deficiency and environmental factors.  **Citation:** Machluf Y, Allon G, Sebbag A, Chaiter Y, Mezer E. A large population study reveals a novel association between congenital color vision deficiency and environmental factors. Graefe's Archive for Clinical and Experimental Ophthalmology. 2022 Apr 1:1-9. | | The study was not conducted in Africa (Israel) | |
| 3.42 | **Title:** Color blindness defect and medical laboratory technologists  **Citation:** Dargahi H, EYN EN, Dashti N. Color blindness defect and medical laboratory technologists: Unnoticed problems and the care for screening. | | The study was not conducted in Africa (Iran) | |
| 3.43 | **Title:** Colour vision deficiency is associated with increased prevalence of amblyopia, strabismus and ametropia  **Citation:** Barayev E, Shapiro M, Greenbaum E, Ran Y, Gershoni A, Dotan G. Colour vision deficiency is associated with increased prevalence of amblyopia, strabismus and ametropia: a large population study. British Journal of Ophthalmology. 2024 Nov 12. | | The study was not conducted in Africa (Israel) | |
| 3.44 | **Title:** Hypertension and color blindness in young men.  **Citation:** Morton WE. Hypertension and color blindness in young men. Archives of Internal Medicine. 1975 May 1;135(5):653-6. | | The study was not conducted in Africa (USA) | |
| 3.45 | **Title:** Congenital Colour Vision Deficiency among Patients Attending Outpatient Department of Ophthalmology in a Tertiary Care Centre: A Descriptive Cross-sectional Study.  **Citation:** Shrestha P, Pradhan PM. Congenital Colour Vision Deficiency among Patients Attending Outpatient Department of Ophthalmology in a Tertiary Care Centre: A Descriptive Cross-sectional Study. JNMA: Journal of the Nepal Medical Association. 2022 Mar;60(247):278. | | The study was not conducted in Africa (Nepal) | |
| 3.46 | **Title:** Prevalence of congenital red-green color vision defects in Arab boys from Riyadh, Saudi Arabia.  **Citation:** Osuobeni EP. Prevalence of congenital red-green color vision defects in Arab boys from Riyadh, Saudi Arabia. Ophthalmic epidemiology. 1996 Jan 1;3(3):167-70. | | The study was not conducted in Africa (Saudi Arabia) | |
| 3.47 | **Title:** Frequencies of different types of colour vision defects in the Netherlands.  **Citation:** De Vries-de Mol EC, Went LN. Frequencies of different types of colour vision defects in the Netherlands. Human Heredity. 1978 Aug 28;28(4):301-16. | | The study was not conducted in Africa (Netherlands) | |
| 3.48 | **Title:** Impact of congenital colour vision defects on occupation.  **Citation:** Cumberland P, Rahi JS, Peckham CS. Impact of congenital colour vision defects on occupation. Archives of disease in childhood. 2005 Sep 1;90(9):906-8. | | The study was not conducted in Africa (United Kingdom) | |
| 3.49 | **Title:** Acquired colour vision deficiency in patients receiving digoxin maintenance therapy.  **Citation:** Lawrenson JG, Kelly C, Lawrenson AL, Birch J. Acquired colour vision deficiency in patients receiving digoxin maintenance therapy. British journal of ophthalmology. 2002 Nov 1;86(11):1259-61. | | The study was not conducted in Africa (United Kingdom) | |
| 3.50 | **Title:** Projected color slides as a method for mass screening test for color vision deficiency (a preliminary study).  **Citation:** GÜNDOGAN NÜ, Durmazlar N, Gümüş K, ÖZDEMİR PG, Altintaş AG, Durur I, Acaroglu G. Projected color slides as a method for mass screening test for color vision deficiency (a preliminary study). International journal of neuroscience. 2005 Jan 1;115(8):1105-17. | | The study was not conducted in Africa (Turkey) | |
| 3.51 | **Title:** The incidence of color-blindness among the Chinese.  **Citation:** Kilborn LG, Beh YT. The incidence of color-blindness among the Chinese. Science. 1934 Jan 12;79(2037):34-. | | The study was not conducted in Africa and lack of accesses | |
| 3.52 | **Title:** On the Incidence of Color-Blindness Among Negroes.  **Citation:** Crooks KB. On the Incidence of Color-Blindness Among Negroes. Science. 1934 Sep 21;80(2073):269-. | | The study is not available on data base during search | |
| 3.53 | **Title:** Congenital color blindness in young Turkish men.  **Citation:** Citirik M, Acaroglu G, Batman C, Zilelioglu O. Congenital color blindness in young Turkish men. Ophthalmic epidemiology. 2005 Jan 1;12(2):133-7. | | The study was not conducted in Africa Turkey) | |
| 3.54 | **Title:** Ocular Diseases Among Pathologists and Pathologists' Perceptions on Ocular Diseases: A Survey Study.  **Citation:** Akman O, Kösemehmetoğlu KE. Ocular Diseases Among Pathologists and Pathologists' Perceptions on Ocular Diseases: A Survey Study. Turk patoloji dergisi. 2015;31. | | The study was not conducted in Africa (Turkey) | |
| 3.56 | **Title:** The incidence of color blindness among races.  **Citation:** Garth TR. The incidence of color blindness among races. Science. 1933 Mar 31;77(1996):333-4. | | The study was not conducted in Africa (USA) | |
| 3.57 | **Title:** The incidence of color-blindness among Jewish males. Science.  **Citation:** Shuey AM. The incidence of color-blindness among Jewish males. Science. 1936 Sep 4;84(2175):228-. | | Both abstract and full articles are unavailable | |
| 3.58 | **Title:** Pattern of ocular morbidities: A cross-sectional study on school-going children in Shillong city**.**  **Citation:** Sarkar A, Medhi GK, Bhattacharyya H, Pala S, Gogoi S. Pattern of ocular morbidities: A cross-sectional study on school-going children in Shillong city. Journal of Family Medicine and Primary Care. 2019 Jun 1;8(6):2124-8. | | The study was not conducted in Africa (India) | |
| 3.59 | **Title:** Incidence of red-green colour blindness in the Basque population.  **Citation:** Rebato E, Calderon R. Incidence of red-green colour blindness in the Basque population. Anthropologischer Anzeiger. 1990 Jun 1:145-8. | | The study was not conducted in Africa (Basque) | |
| 3.6 | **Title:** Prevalence of congenital color deficiencies in secondary-school students in Tehran.  **Citation:** Modarres M, Mirsamadi M, Peyman GA. Prevalence of congenital color deficiencies in secondary-school students in Tehran. International ophthalmology. 1996 Jul;20:221-2. | | The study was not conducted in Africa (Iran) | |
| 3.61 | **Title:** Color vision deficiencies in youths 12-17 years of age, United States.  **Citation:** Slaby D, Roberts J. Color vision deficiencies in youths 12-17 years of age, United States. National Center for Health Statistics; 1974. | | The study was not conducted in Africa (USA) | |
| 3.62 | **Title:** Blood groups, ABH saliva secretion and colour vision deficiency in Hindu castes and religious groups of West Godavari, Andhra Pradesh, India.  **Citation:** Vijayalakshmi M, Naidu JM, Suryanarayana B. Blood groups, ABH saliva secretion and colour vision deficiency in Hindu castes and religious groups of West Godavari, Andhra Pradesh, India. Anthropologischer Anzeiger. 1994 Dec 1:305-13. | | The study was not conducted in Africa (India) | |
| 3.63 | **Title:** Screening for congenital colour vision defects: A comparison between the Ohkuma and Ishihara plates.  **Citation:** Littlewood R, Hyde F. Screening for congenital colour vision defects: A comparison between the Ohkuma and Ishihara plates. Australian and New Zealand Journal of Ophthalmology. 1993 Feb;21(1):31-5. | | The study was not conducted in Africa (Australia) | |
| 3.64 | **Title: Incidence of color blindness: green and red.**  **Citation:** Goncerzewicz M, Krawczyński M, Suchocka-Luczak S. Incidence of color blindness: green and red. Wiadomosci Lekarskie (Warsaw, Poland: 1960). 1971 Apr 1;24(7):639-43. | | The article is not available in English and the study was not conducted in Africa (Poland) | |
| 3.65 | **Title:** Retinitis pigmentosa and color vision deficiency in Kamigoto Island, Nagasaki Prefecture.  **Citation:** Toda S. Retinitis pigmentosa and color vision deficiency in Kamigoto Island, Nagasaki Prefecture. Nippon Ganka Gakkai Zasshi. 1997 Aug 1;101(8):669-76. | | The study was not conducted in Africa (Japan). The article was published in Japanese and it’s not accessible | |
| 3.66 | **Title:** Assessing for colour vision defects.  **Citation:** Ellis D. Assessing for colour vision defects. Special Education: Forward Trends. 1981 Sep 1;8(3):15-. | | Abstract is not available and the article is not freely accessible | |
| 3.67 | **Title:** The Incidence of Color Blindness Among Deaf Children.  **Citation:** Frey RM, Krause IB. The Incidence of Color Blindness Among Deaf Children. Exceptional Children. 1971 Jan 1;37(5). | | Abstract is not available and the article is not freely accessible | |
| 3.68 | **Title:** The incidence of congenital disorders of the red-green image in the Persian Azerbaijan.  **Citation:** PLATTNER F. The incidence of congenital disorders of the red-green image in the Persian Azerbaijan. Albrecht von Graefe's Archiv fur Ophthalmologie. 1959;161:237-8. | | the study was not conducted in Africa (German), and lacks abstract | |
| 3.69 | **Title:** Red-green colour blindness in the Tormes-Alberche Valley (Avila-Central Spain).  **Citation:** Cabrero FJ, Ortiz MA, Mesa MS, Fuster V, Moral P. Red-green colour blindness in the Tormes-Alberche Valley **(Avila-**Central Spain). Anthropol Anz. 1997 Dec;55(3-4):295-301. PMID: 9468757. | | the study was not conducted in Africa (Spain) | |
| 3.70 | **Title:** Ocular findings among young men: a 12-year prevalence study of military service in Poland  **Citation:** Nowak MS, Jurowski P, Gos R, Smigielski J. Ocular findings among young men: a 12‐year prevalence study of military service in Poland. Acta ophthalmologica. 2010 Aug;88(5):535-40. | | The study was not conducted in Africa (Poland) | |
| 3.71 | **Title:** Color vision defects in school children.  **Citation:** Verd Vallespir S, Martínez López M. Defectos de visión de los colores en el ámbito de la medicina escolar [Color vision defects in school children]. An Esp Pediatr. 1991 Nov;35(5):309-12. Spanish. PMID: 1785743. | | Abstract is not available and full text is not accessible | |
| 3.72 | **Title:** Ocular morbidity prevalence among school children in Shimla, Himachal, North India.  **Citation:** Gupta M, Gupta BP, Chauhan A, Bhardwaj A. Ocular morbidity prevalence among school children in Shimla, Himachal, North India. Indian J Ophthalmol. 2009 Mar-Apr;57(2):133-8. doi: 10.4103/0301-4738.45503. PMID: 19237787; PMCID: PMC2684438. | | The study was not conducted in Africa (India) | |
| 3.73 | **Title: Colour vision defects acquired in diseases of the eye.**  **Citation:** COX J. Colour vision defects acquired in diseases of the eye. Br J Physiol Opt. 1961 Apr;18:67-79 concl. PMID: 13696130. | | Abstract and article is not accessible | |
| 3.74 | **Title:** A visual profile of Queensland Indigenous children.  **Citation:** Hopkins S, Sampson GP, Hendicott PL, Wood JM. A visual profile of Queensland Indigenous children. Optometry and Vision Science. 2016 Mar 1;93(3):251-8. | | The study was not conducted in Africa (Australia) | |
| 3.75 | **Title:** Colour vision deficiency: the ‘unseen’disability.  **Citation:** Webster B. Colour vision deficiency: the ‘unseen’disability. British Journal of Nursing. 2021 Apr 22;30(8). | | Abstract is not available and article is not freely accessible | |
| 3.76 | **Title**: Diagnosis of colour-vision defects in very young children. **Citation:** Sassoon H, Wise J. Diagnosis of colour-vision defects in very young children. The Lancet. 1970 Feb 21;295(7643):419-20. | | Abstract is not available and article is not accessible | |
| 3.77 | **Title:** Diagnosis of colour-vision defects in very young children.  **Citation:** Taylor WO. Diagnosis of colour-vision defects in very young children. The Lancet. 1970 Apr 11;295(7650):781-2. | | Abstract is not available and article is not accessible | |
| 3.78 | **Title:** Acquired color vision deficiency.  **Citation:** Simunovic MP. Acquired color vision deficiency. Survey of ophthalmology. 2016 Mar 1;61(2):132-55. | | The article does not contain the outcome of interest and it’s a review | |
| 4. | Full-text article assessment performed | | 26 | |
| 5 | Included studies after Full-text article assessment | | 16 | |
| 5.1 | **Authors:** Gudeta and Asrat et al., [12]  **Topic:** Prevalence and genotypic frequency of color vision defects among primary schoolchildren in Adama Town, Eastern Ethiopia  **Citation:** Gudeta TB, Asrat T. Prevalence and genotypic frequency of color vision defects among primary schoolchildren in Adama Town, Eastern Ethiopia. BMC pediatrics. 2024;24(1):72. | | | |
| 5.2 | **Authors:** Darge et al., [24]  **Topic:** The Prevalence of Visual Acuity Impairment among School Children at Arada Subcity Primary Schools in Addis Ababa  **Citation:** Darge HF, Shibru G, Mulugeta A, Dagnachew YM. The Prevalence of Visual Acuity Impairment among School Children at Arada Subcity Primary Schools in Addis Ababa, Ethiopia. Journal of ophthalmology. 2017;2017:9326108 | | | |
| 5.3 | **Authors:** Mashige et al., [25]  **Topic:** Impact of congenital color vision defect on color-related tasks among schoolchildren in Durban, South Africa  **Citation:** Mashige KP. Impact of congenital color vision defect on color-related tasks among schoolchildren in Durban, South Africa. Clinical optometry. 2019;11:97-102. | | | |
| 5.4 | **Authors:** Dohvoma et al., [26]  **Topic:** Color vision deficiency among biomedical students: a cross-sectional study  **Citation:** Dohvoma VA, Ebana Mvogo SR, Kagmeni G, Emini NR, Epee E, Mvogo CE. Color vision deficiency among biomedical students: a cross-sectional study. Clinical ophthalmology (Auckland, NZ). 2018;12:1121-4. | | | |
| 5.5 | **Authors:** Wale et al., [27]  **Topic:** Prevalence of color blindness among school children in three primary schools of Gish -Abay town district, Amhara regional state, north-west Ethiopia.  **Citation:** Wale MZ, Abebe Y, Adamu Y, Zelalem A. Prevalence of color blindness among school children in three primary schools of Gish -Abay town district, Amhara regional state, north-west Ethiopia. BMC ophthalmology. 2018;18(1):306. | | | |
| 5.6 | **Authors:** Mitiku et al, [28]  **Topic:** Prevalence and allele frequency of Congenital Colour Vision Deficiency (CCVD) among students at Hawassa University, Ethiopia.  **Citation:** Mitiku RG, Tolera BS, Tolesa ZG. Prevalence and allele frequency of Congenital Colour Vision Deficiency (CCVD) among students at Hawassa University, Ethiopia. The Journal of the Egyptian Public Health Association. 2020;95(1):10. | | | |
| 5.7 | **Authors:** Ugalahi et al., [29]  **Topic:** Prevalence of congenital colour vision deficiency among secondary school students in Ibadan, South-West Nigeria  **Citation:** Ugalahi MO, Fasina O, Ogun OA, Ajayi BG. Prevalence of congenital colour vision deficiency among secondary school students in Ibadan, South-West Nigeria. The Nigerian postgraduate medical journal. 2016;23(2):93-6. | | | |
| 5.8 | **Authors:** Oduntan et al.,[17]  **Topic:** Colour vision deficiency among students in Lagos State, Nigeria. African health sciences  **Citation:** Oduntan OA, Mashige KP, Kio FE. Colour vision deficiency among students in Lagos State, Nigeria. African health sciences. 2019;19(2):2230-6 | | | |
| 5.9 | **Authors:** Tabansi et al., [30]  **Topic:** Screening for congenital color vision deficiency in primary children in Port Harcourt City; teachers' knowledge and performance  **Citation:** Tabansi PN, Anochie IC, Nkanginieme KE, Pedro-Egbe CN. Screening for congenital color vision deficiency in primary children in Port Harcourt City; teachers' knowledge and performance. Nigerian journal of medicine : journal of the National Association of Resident Doctors of Nigeria. 2008;17(4):428-32. | | | |
| 5.10 | **Authors:** Woldeamanuel et al., [18]  **Topic:** Prevalence of color vision deficiency among school children in Wolkite, Southern Ethiopia  Citation: Woldeamanuel GG, Geta TG. Prevalence of color vision deficiency among school children in Wolkite, Southern Ethiopia. BMC research notes. 2018;11(1):838. | | | |
| 5.11 | **Authors:** Mulusew et al., [31]  **Topic:** Prevalence of congenital color vision defects among school children in five schools of Abeshge District, Central Ethiopia.  **Citation:** Asferaw M, A Y. Prevalence of congenital color vision defects among school children in five schools of Abeshge District, Central Ethiopia. Journal of Ophthalmology of Eastern, central and southern Africa. 2013;17:10-4. | | | |
| 5.12 | **Authors:** Eze et al., [4]  **Topic:** Colour vision defect among secondary school students in enugu, Nigeria  **Citation:** Eze GC, Kizor-Akaraiwe N, Chime AA, Anajekwu CC, Asimadu IN, Edoga CE, et al. Colour vision defect among secondary school students in enugu, Nigeria: prevalence, pattern and impact. Advance in Ophthalmology & Visual System. 2020;10(5):113-9. | | | |
| 5.13 | **Authors:** Mengesha et al., [32] **(Preprint study)**  **Topic:** Prevalence and Allele Frequency of Red-Green Color Vision Defects among School Children in Repi Primary School in Addis Ababa, Ethiopia. 2021.  **Citation:** Mengesha WA, Mengistu AG, Tolesa ZG. Prevalence and Allele Frequency of Red-Green Color Vision Defects among School Children in Repi Primary School in Addis Ababa, Ethiopia. 2021.  **Preprint studies(Avaliable at):** <https://www.researchgate.net/publication/352534342> | | | |
| 5.14 | **Authors:** Fakorede et al, [33]  **Topic:** Prevalence and population genetic data of colour vision deficiency among students from selected tertiary institutions in Lagos State, Nigeria  **Citation:** Fakorede S, Akpan L, Adekoya K, Oboh B. Prevalence and population genetic data of colour vision deficiency among students from selected tertiary institutions in Lagos State, Nigeria. Egyptian Journal of Medical Human Genetics. 2022;23 | | | |
| 5.15 | **Authors:** Ativie et al., [34]  **Topic:** Prevalence of Congenital Colour Vision Deficiency in Nigerians Living in Ugep, Cross River State  **Citation:** Ativie R, Ubom R, Aigbiremolen A, Mukoro O, Odigie O, Igweh J. Prevalence of Congenital Colour Vision Deficiency in Nigerians Living in Ugep, Cross River State. Ophthalmology Research: An International Journal. 2017;7:1-6.  35. | | | |
| 5.16 | **Authors:** Nwobodo et al., [35]  **Topic:** Prevalence of Colour Blindness in Nigeria: Nnamdi Azikiwe University Medical  **Citation:** Nwobodo E, Nneoma D, Ikwuka D. Prevalence of Colour Blindness in Nigeria: Nnamdi Azikiwe University Medical. Cyprus Journal of Medical Sciences. 2021. | | | |
| 6 | Excluded studies after full text article assessment (N=10) | | | Reason(s) for exclusion |
| 6.1 | **Authors: Geletu et al., 2018**  **Topic:** Identification of colorblindness among selected primary school children in Hararghe Region, Eastern Ethiopia  **Citation:** Muthuswamy M, Oljira T, Geletu T. Identification of colorblindness among selected primary school children in Hararghe Region, Eastern Ethiopia. Alexandria Journal of Medicine. 2018;54. | | | The study does not meet minimum quality assessment (high risk of Bias) |
| 6.2 | **Authors: Geletu et al., 2018, Pickford and Pickford, 1981, Rahman et al., 1998, Rosa, 1981, Osman et al., 2021**  **Topic:** Prevalence and predictors of colour vision defects among Egyptian university students  **Citation:** Osman S, Khalaf S, Mohammed H, El-Sebaity D, Osman D. Prevalence and predictors of colour vision defects among Egyptian university students. Eastern Mediterranean health journal = La revue de sante de la Mediterranee orientale = al-Majallah al-sihhiyah li-sharq al-mutawassit. 2021;27(4):399-406. | | | The study does not meet minimum quality assessment (high risk of Bias) |
| 6.3 | **Authors: Pickford and Pickford, 1981**  **Topic:** Frequency of colour vision defects among Zulus in Natal  **Citation:** Pickford RW, Pickford R. Frequency of colour vision defects among Zulus in Natal. Journal of biosocial science. 1981;13(2):241-8. | | | The study does not meet minimum quality assessment (high risk of Bias) |
| 6.4 | **Authors: Rahman et al., 1998**  **Topic:** Comparison of the incidence of colour blindness between sections of Libyan and Indian populations.  **Citation:** Rahman SA, Singh PN, Nanda PK. Comparison of the incidence of colour blindness between sections of Libyan and Indian populations. Indian journal of physiology and pharmacology. 1998;42(2):271-5. | | | The study does not meet minimum quality assessment (high risk of Bias) |
| 6.5 | **Authors: Rosa, 1981**  **Topic:** The Distribution of Red and Green Colourblindness in Kenya  **Citation:** Rosa PJM. The Distribution of Red and Green Colourblindness in Kenya. Environmental Science 1981:286-99. | | | The study does not meet minimum quality assessment (high risk of Bias) |
|  | **Authors: Borges da Silva, 1983,**  **Topic:** The prevalence of color blindness in Senegal in a population of 4500 workers  **Citation:** Borges da Silva G, Gabaye Borges da Silva G. The prevalence of color blindness in Senegal in a population of 4500 workers]. Bulletin de la Societe de pathologie exotique et de ses filiales. 1983;76(5 Pt 2):841-5 | | | Lack of Full text article access |
| 6.5 | **Authors: Odeigah and Okon, 1986**  **Topic:** Colour vision defects and gene flow in Nigerians. East African medical journal  **Citation:** Odeigah PG, Okon EE. Colour vision defects and gene flow in Nigerians. East African medical journal. 1986;63(10):666-71 | | | Lack of Full text article access |
| 6.6 | **Authors: Ohta et al., 1978**  **Topic:** Clinical analysis of colour vision deficiencies with The City University test.  **Citation:** Ohta Y, Kogure S, Izutsu Y, Miyamoto T, Nagai I. Clinical analysis of colour vision deficiencies with The City University test. Modern problems in ophthalmology. 1978;19:126-30. | | | Lack of Full text article access |
| 6.7 | **Authors: Mashige and Van Staden, 2019**  **Topic:** Prevalence of congenital colour vision deficiency among Black school children in Durban, South Africa.  **Citation:** Mashige KP, van Staden DB. Prevalence of congenital colour vision deficiency among Black school children in Durban, South Africa. BMC research notes. 2019;12(1):324.  47. | | | Duplication found (similar studies were published using different title) |
| 6.8 | **Authors: Zelalem et al., 2019**  **Topic:** Prevalence of visual impairment among school children in three primary schools of Sekela Woreda, Amhara regional state, north-west Ethiopia.  **Citation:** Zelalem M, Abebe Y, Adamu Y, Getinet T. Prevalence of visual impairment among school children in three primary schools of Sekela Woreda, Amhara regional state, north-west Ethiopia. SAGE open medicine. 2019;7:2050312119849769. | | | Duplication found (similar studies were published using different title) |
